# Optimized and Personalized Phlebotomy Schedules for Patients Suffering From Polycythemia Vera

**DOI:** 10.3389/fphys.2020.00328

**Published:** 2020-04-17

**Authors:** Patrick Lilienthal, Manuel Tetschke, Enrico Schalk, Thomas Fischer, Sebastian Sager

**Affiliations:** ^1^Institute for Mathematical Optimization, Otto-von-Guericke-University Magdeburg, Magdeburg, Germany; ^2^Department of Hematology and Oncology, Medical Center, Otto-von-Guericke-University Magdeburg, Magdeburg, Germany

**Keywords:** polycythemia vera, optimal control, modeling, numerical simulation, therapy scheduling, mixed-integer non-linear optimization, cancer, decision support

## Abstract

Polycythemia vera (PV) is a slow-growing type of blood cancer, where the production of red blood cells (RBCs) increase considerably. The principal treatment for targeting the symptoms of PV is bloodletting (phlebotomy) at regular intervals based on data derived from blood counts and physician assessments based on experience. Model-based decision support can help to identify optimal and individualized phlebotomy schedules to improve the treatment success and reduce the number of phlebotomies and thus negative side effects of the therapy. We present an extension of a simple compartment model of the production of RBCs in adults to capture patients suffering from PV. We analyze the model's properties to show the plausibility of its assumptions. We complement this with numerical results using exemplary PV patient data. The model is then used to simulate the dynamics of the disease and to compute optimal treatment plans. We discuss heuristics and solution approaches for different settings, which include constraints arising in real-world applications, where the scheduling of phlebotomies depends on appointments between patients and treating physicians. We expect that this research can support personalized clinical decisions in cases of PV.

## 1. Introduction

The disease polycythemia vera (PV) belongs to chronic myeloproliferative neoplasms, meaning that an excess of blood cells are produced. In particular, red blood cells (RBCs) are affected (Lichtman et al., 2006). With an increasing number of RBCs in the human body, there is increased risk of thromboembolic events (Marchioli, 2005). To prevent patients from suffering serious events, such as strokes, heart attacks, or pulmonary embolisms, the density of the blood must be reduced. In moderate cases of the disease, this can be achieved with blood-letting (phlebotomy) at regular intervals (Tefferi et al., 2018).

In those cases, therapy schedules based on blood image data are proposed by physicians. However, those schedules might not be optimal for each individual (Finazzi and Barbui, 2007). These patients benefit considerably from a therapeutic strategy, that is able to predict the optimal treatment time for the next phlebotomy. In this paper, therefore, the data-driven model for erythropoiesis by Tetschke et al. (2018), verified for use on the data of healthy subjects, is extended to include amplified cell production by PV. Model analysis is applied to derive properties that emphasize the model's plausibility for this disease. Clinical data from PV patients and *in silico* data derived from healthy subjects are used to evaluate and compare different optimization strategies for computing individual patient treatment schedules. Such strategies are for the most part capable of including constraints that appear in clinical applications, including reasonable clinical treatment times.

Using our results, it might be possible to enable physicians to schedule therapies individually based on a set of parameters unique to each patient. Thus, on the one hand, the probability of severe complications will decrease, when the time until the next measurement is assumed to be too long. On the other hand, in cases where the frequency of two consecutive measurements is assumed to be too low, the patient will benefit from not needing to go to a hematologist and the patient will be spared additional blood withdrawals.

To our knowledge there is neither a published mathematical model of erythropoiesis, that considers the disease PV, nor a study discussing optimal treatment schedules for PV patients by phlebotomy.

The paper is organized as follows: first, in chapter 2, we present the materials and methods used for this research. In chapter 3 we display the results of the modeling and the optimization approaches. Finally, we summarize and discuss our findings in chapter 4. Given the interdisciplinary nature of this research project, literature surveys are included in the corresponding subsections of this paper.

## 2. Materials and Methods

In this section, we present the concepts and methods for modeling PV and for computing optimal treatment schedules. First, biological properties necessary for the modeling process are summarized. Then, a published compartment model for erythropoiesis in healthy subjects is reviewed. Afterwards, the acquisition of data from real and artificial patients is presented. Finally, computational methods for verifying the proposed model and for generating treatment schedules are discussed.

### 2.1. Biological Background

Understanding the relevant biological processes is crucial for the following modeling process. To this end, basic information about the physiological processes of erythropoiesis and of PV are summarized in this section.

#### 2.1.1. Summary of Erythropoiesis

The supply of oxygen from the lungs to tissues and the transport of carbon dioxide back from tissues is central for the maintenance of vital functions in the human body. This exchange of substances is realized by erythrocytes (i.e., RBCs), which are biconcave discoid cells in the blood stream containing the protein complex hemoglobin. This protein complex binds the substances and enables the RBCs to their part. At any given time, a healthy adult human has a total of 2–3·10^13^ erythrocytes, with men and women having about 5–6 million and 4–5 million erythrocytes per microliter of blood, respectively.

**Figure 1:**
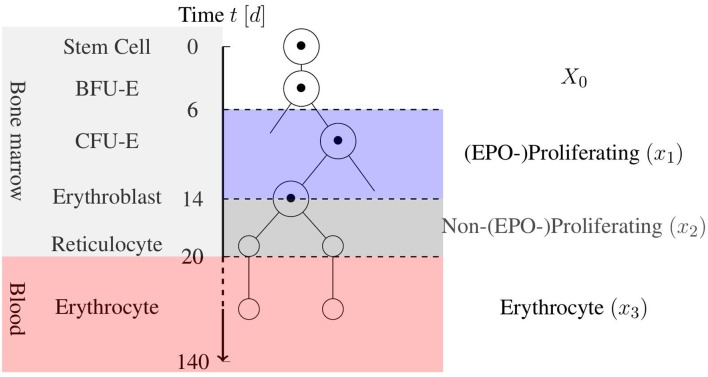
Simplified schematic view of erythropoiesis. Certain cell stages over the age of the cell in days are displayed with a corresponding cell partition based on the model by Tetschke et al. (2018).

Erythropoiesis is the process by which RBCs are produced in the bone marrow. Beginning with stem cells, multi-potent stem cells are matured through several levels of erythroid progenitor cells, i.e., the Blast Forming Unit-Erythroid (BFU-E) and Colony Forming Unit-Eryhroid (CFU-E), and several levels of erythroblasts to bone marrow reticulocytes. These are then released into the blood circulation as blood reticulocytes, which then quickly grow into mature erythrocytes. During this process, which takes ~20 days, the cell undergoes major changes including the removal of nuclei, organelles, and mitochondria to provide more room for hemoglobin. This process is displayed in Figure 1 in a simplified scheme. The mature RBC has no nucleus, and it is incapable of cell division and regeneration of cell tissue. Damaged cells are removed by phagocytes to prevent clogging. This determines the mean life expectancy of RBCs in the blood stream, which is ~120 days in healthy adults (Jandl, 1987). Sufficient iron concentration in the blood stream is necessary for successful erythropoiesis.

The hormone erythropoietin (EPO) is mainly responsible for the response of the body to changes in the amount of RBCs. It acts like a negative feedback mechanism for erythropoiesis. The EPO concentration in the blood circulation is inversely related to the concentration of hemoglobin. High EPO concentrations result in an increase to the RBC proliferation rate in the bone marrow. Several precursor cell types are affected, especially CFU-E production. This short summary can be complemented by a more detailed overview of erythropoiesis in Lichtman et al. (2006).

#### 2.1.2. The Disease Polycythemia Vera

Polycythemia vera, also called primary polycythemia, is a chronic myeloproliferative neoplasm. That is, the production of blood cells increase to pathological levels. Most prominently, erythrocytes (i.e., RBCs) are affected. This causes the main symptoms of the patients: if the ratio of erythrocytes to the total blood volume—which, in medical terms, is called the hematocrit (*Hct*)—exceeds a certain threshold, the blood cells can clot. This can cause thromboembolic events, which can lead to strokes, myocardial infarctions, vein/arterial thrombosis, or pulmonary embolisms. These events can also often be located in atypical sides (Kiladjian et al., 2008; Dentali et al., 2014). While RBCs are mainly responsible for the clotting, also leukocytes and platelets as well as inflammatory mechanisms have an impact on the thromboembolic events (Falanga and Marchetti, 2014; Koschmieder et al., 2016).

If untreated, the mean life expectancy of patients suffering from PV is only ~18 months (Marchioli, 2005; Lichtman et al., 2006). On the other hand, with treatment, a normal life span can be assumed (c.f. Rozman et al., 1991).

Other symptoms of the disease are not fatal, but can strongly reduce the quality of life of the patient. Most prominently, aquagenic pruritus, a severe itching that patients experience from contact with water, is observed in up to 70% of cases (Siegel et al., 2013). Furthermore, patients suffer from headaches, hypertonia, fatigue, weight loss, and night sweats (Policitemia, 1995; Scherber et al., 2011). Also splenomegaly can be observed in PV patients. As described in Marchioli (2005), PV patients have a higher risk of developing other types of blood cancer over time, such as acute myeloid leukemia or myelofibrosis. This risk is associated with the age of the patient and the duration of the disease. After eight years, the disease evolves into secondary post-polycythaemic myelofibrosis in 15% of the cases (35% after 15 years, c.f. Alvarez-Larrán et al., 2009). In 20% of these cases, the patients develop acute myeloid leukemia (Mesa et al., 2005).

In low-risk cases, the basic therapy for PV is blood-letting (phlebotomy): ~500 ml of blood on a regular basis (Tefferi et al., 2018). As the body is compensating for blood loss through blood plasma within a short amount of time yet requires several weeks to produce new RBCs, the *Hct* can be temporarily reduced using this treatment. In severe cases, this procedure is insufficient and there is the need for cytoreductive therapy (or a combination of both). It is currently unknown, how the frequency and volume of phlebotomies should be calculated to give an optimal outcome for the patient (Marchioli, 2005).

The most important clinical parameter for the planning of the treatment ist *Hct*. Additionally, counts of leukocytes, platelets, size of the spleen and other symptoms are taken into account (Barbui et al., 2011, 2018). In clinical practice, a phlebotomy is executed in a PV patient if the *Hct* is above 45% (Lichtman et al., 2006). According to Finazzi and Barbui (2007), this threshold might be inappropriate, because these findings were based on retrospective studies with small sample sizes and methodological shortcomings. They were unable to associate severe implications with *Hct* values between 40 and 55% in a larger prospective study. Contrarily, in a more recent study (Marchioli et al., 2013) showed that the rate of major thromboembolic events was significantly higher, if a target *Hct* of 45–50% was used. They recommend a target *Hct* of below 45%. Due to these conflicting results, the complementation of the *Hct* treatment criterion by additional information regarding individual patients might yield additional insights. To the best of our knowledge, no such approach to doing so exists.

The regulation of erythropoiesis no longer works in patients suffering from PV. The underlying process has yet to be fully understood, although there are plausible assumptions about it. In the investigation by Eaves and Eaves (1978), it was observed that in PV patients there is a partition in the CFU-E population. In the first fraction of cells, EPO exerts a normal influence when controlling the population, and in the second fraction, the cells proliferate unbounded, even at extremely low levels of EPO. In most (but not all) PV patients, a mutation of the JAK2V617F gene is present (Pardanani et al., 2007). This is associated with an uncontrolled proliferation of the progenitor cells (Lichtman et al., 2006). However, the direct influence of the mutation on erythropoiesis in PV is not fully understood. The JAK2V617F allele burden, i.e., the fraction of genes affected by that mutation, can be measured. More thorough understanding of JAK mutations has recently led to an increasing influence on therapy decisions in other hematopoietic diseases (Vainchenker et al., 2008). However, it does not seem to have a direct impact on Hct or the number of treatments (Silver et al., 2011).

### 2.2. Data-Driven Model for Erythropoiesis in Healthy Subjects

A mathematical model of erythropoiesis in healthy adults was developed in Tetschke et al. (2018). This simple compartment model focuses on the system dynamics after blood loss, and it should be capable of capturing the relevant mechanisms in the case of a phlebotomy in a PV patient. Using the model, a suitable choice of model parameters was made such that the model reflected the subjects individually. The simulation results using this parameter set were verified using high-quality clinical data. In addition, the identifiability of the model parameters was positively investigated.

Basically, the model consists of three ordinary differential equations, that characterize the maturation and differentiation of a stem cell into an RBC until its death. Instead of incorporating EPO directly, the model uses an indirect approach with the help of the feedback function *Fb*(·). Thus, a decrease in the number of RBCs in *x*_3_ results in an increased proliferation in *x*_1_.

The three compartment model for erythropoiesis by Tetschke et al. (2018) is given by

(1) $$\begin{array}{l} {ẋ}_{1}\left(t\right)=\beta \cdot \left(X_{0}-k_{1}\cdot x_{1}\left(t\right)\right)+Fb\left(x_{3}\left(t\right)\right)\cdot x_{1}\left(t\right)\\ \begin{array}{c} {ẋ}_{2}\left(t\right)=\beta \cdot \left(k_{1}\cdot x_{1}\left(t\right)-k_{2}\cdot x_{2}\left(t\right)\right) \end{array}\\ \begin{array}{c} {ẋ}_{3}\left(t\right)=\beta \cdot \left(k_{2}\cdot x_{2}\left(t\right)-\alpha \cdot x_{3}\left(t\right)\right)\\ Fb\left(x_{3}\left(t\right)\right)=\gamma \cdot \left(1-\frac{x_{3}\left(t\right)}{B}\right) \end{array} \end{array}$$

with the following model components:

- The compartments *x*_1_ [1] and *x*_2_ [1] reflect certain precursor cells in the bone marrow, that are committed to the erythrocyte lineage. *x*_1_ includes CFU-E and early erythroblasts, which are highly affected by EPO in the blood circulation. *x*_2_ denotes late erythroblasts and reticulocytes, which are unaffected or only slightly affected by EPO.
- The compartment *x*_3_ [*g*] contains the mass of mature erythrocytes in the blood stream.
- *X*_0_ [*d*^−1^] denotes a constant inflow from the stem cell compartment into the erythroid lineage.
- β [1] is a factor for EPO-independent proliferation. This is assumed to be unique to the patient.
- γ [*d*^−1^] is a factor for EPO-dependent proliferation of early precursor cells. This is also assumed to be unique to the patient.
- *k*_1_ [*d*^−1^], *k*_2_ [*d*^−1^] and α [(*gd*)^−1^] are the transition and apoptosis rates given by the literature (Tetschke et al., 2018). It remains unclear whether these transition rates are dependent on EPO. Here, they are assumed to be EPO-independent and set to $\frac{1}{8}$, $\frac{1}{6}$, and $\frac{1}{120}$, respectively, based on the literature values.
- In the case of healthy erythropoiesis, the existence of an average normal erythrocyte level can be assumed, when environmental conditions do not change drastically. The average value is denoted by *B* [*g*].
- *Fb*(·) [1] is a negative feedback function based on the fractional loss in *x*_3_, meaning, that the function decreases with increasing values of *x*_3_ and vice versa. This indirectly incorporates the EPO dependency of the first compartment. By only using this function as a feedback, it was implicitly assumed that this is the only proliferation amplification factor from blood loss. This assumption is reasonable, provided that the blood loss is not too high, as, for example, in the case of severe where anemia emergency reactions like the release of stress reticulocytes (Lichtman et al., 2006) occur.

Blood removal of at most *V*_max_ *ml* of blood can be realized in a discrete way by removing $u\left(t\right)\cdot \frac{V_{\text{max}}}{V_{\text{pat}}}\cdot x_{3}$ from the third compartment or in a continuous way by modifying the equation for ẋ_3_:

(2) $$\begin{array}{l} {ẋ}_{3}\left(t\right)=\beta \cdot \left(k_{2}\cdot x_{2}\left(t\right)-\alpha \cdot x_{3}\left(t\right)\right)-u\left(t\right)\cdot \frac{V_{\text{max}}}{V_{\text{pat}}}\cdot x_{3}\left(t\right) \end{array}$$

Here, *V*_pat_ is the subject's total blood volume in *ml*, and *u*(*t*) ∈ [0, 1] accounts for the application of (fractional) blood removal. The unique steady state of (1) was shown to be

(3) $$\begin{array}{l} \overset{-}{x}:=\left({\overset{-}{x}}_{1},{\overset{-}{x}}_{2},{\overset{-}{x}}_{3}\right)=\left(\frac{k_{1}}{\alpha },\frac{k_{2}}{\alpha },1\right)\cdot B \end{array}$$

given that *x*_1_, *x*_2_ and *x*_3_ are positive and *X*_0_: = α·*B*.

The model was verified using data from Pottgiesser et al. (2008). There, blood loss of 500 ml in healthy adult subjects with sufficient iron concentrations was taken into account. In Tetschke et al. (2018), sufficient data from one re-saturation cycle after a blood donation could personalize the variables β and γ of the model. The estimation of *B* further improves the quality of the estimations, but in most cases this was not possible, as more data was needed. Details regarding model assumptions, clinical data, and numerical results can be found in Tetschke et al. (2018).

### 2.3. Data

The clinical parameter *Hct*, which is used to determine necessary treatment in clinical practice, suffers from serious drawbacks in measurements. This is mainly from plasma volume deviations, which can be significant in short amounts of time (Pottgiesser et al., 2008; Otto et al., 2017). Further, *Hct* only reflects a relative amount of solid blood particles. Rather, absolute values are needed to compute the effect of phlebotomies.

Indeed, our models need to take into account the absolute amount of erythrocytes in the body. As blood counts only provide information relative to the withdrawn amount, the total blood volume is needed for this computation. As described by Ertl et al. (2007), most measurement techniques for blood volume are invasive, and formulae for such estimations are imprecise. Thus, in Tetschke et al. (2018) the total hemoglobin mass (*tHb*) was used, which indirectly reflects the absolute amount of erythrocytes. This is advantageous insofar as much more accurate measurements can be made. In what follows, we use *tHb*, rather than *Hct* or the number of erythrocytes.

#### 2.3.1. Clinical Data

In cooperation with the Department of Hematology and Oncology at the University Hospital Magdeburg, Germany, we retrospectively collected data from patients suffering from PV. The institutional ethics committee at the University of Magdeburg endorsed the study procedures. Each subject gave written informed consent before participation in this study. Unfortunately, the data were gathered according to routine clinical practice, meaning the quality of the data for use in an optimization study was poor: when treating patients, physicians aim to see patients only when necessary. Thus, the density of the data was quite low. Moreover, only standard blood counts are regularly conducted. Such data suffers the effect of plasma volume deviations and corresponding measurement errors, as described above. Another problem arises with treatment. Phlebotomy is the method of choice, as long as the disease is not too severe. In severe cases, additional therapies with drugs are adopted. For specific medication, a model of the pharmacokinetics and pharmacodynamics of the drug would be helpful. This is beyond the scope of this study, however.

Ultimately, we were able to identify three patient data sets with a reasonable data density and quantity. In Table 1 details about the three patients are displayed. Available data included the relative number of erythrocytes (*Ery* in [*Tpt*/*l*]), the mean corpuscular hemoglobin (*MCH* in [*pg*]), and covariates like the height, weight, and sex of the patient. With the help of Nadler's formula (Nadler et al., 1962) an (error-prone) estimation of the total blood volume in [*l*] was made. Then, *tHb* was computed as the product of *Ery*, *MCH*, and the total blood volume. We excluded data gathered in cases where the patient started a complementary therapy with drugs.

**Table 1:** Details about three clinical patients used in 2.3.1.

**ID**	**Sex**	**Age**	**Time since diagnosis (years)**	**Treatment**
01	Male	45	6	Phlebotomy, chemotherapy since 5 years
02	Female	45	9	Phlebotomy only
03	Female	79	15	Phlebotomy, chemotherapy since 3 years

As many patients are treated for several years, two of the three data sets cover more than five years. One of the assumptions of the model in Tetschke et al. (2018) was that subject-individual parameters are only valid for a certain amount of time. Thus, entire data sets should not be inspected. Instead, we identified periods of time during which there were no drastic changes. This was achieved with change-point analysis and the so-called moving-sum approach by Cho et al. (2018).

#### 2.3.2. Generation of *in silico* Test Data

Owing to the described problems arising from the collection of clinical data, we used data from Pottgiesser et al. (2008) and the resulting parameter sets β and γ obtained in Tetschke et al. (2018), based on a prospective study with 29 healthy adult male subjects using a measurement technique for obtaining *tHb* measurements. Of the data, 28 data sets were used, as one set was excluded in Tetschke et al. (2018).

For the artificial generation of parameters for PV patients from those of healthy subjects, the rejection sampling method (von Neumann, 1963) was used to obtain suitable λ_PV_. These λ_PV_ are suitable, if treatments are necessary and possible with reasonable frequency. For that, a random λ_PV_ was drawn from a uniform distribution on [0, 1]. With the heuristic approach without constraints 2.4.2, a number of necessary treatments within 365 days is generated. A λ_PV_ where that number of treatments is in [1, 26] is accepted. Otherwise, the value is rejected. The interpretation is that the PV patient should be so much affected by the disease that treatment with phlebotomy at least once in a year is necessary. However, it should not be needed more often than twice a month. For patients that are even more sick, physicians proceed with chemotherapy anyway. This process was repeated until, for each subject, five distinct λ_PV_ were found.

This process yielded 140 artificially generated parameter sets of PV patients. The generated values for the five λ_PV_ for each subject were on average in the interval [0.34(±0.12), 0.6(±0.16)] with an overall average number of treatments of 15.56 ± 6.56. The subject parameters with generated λ_PV_ can be found in Table 2.

**Table 2:** Parameter sets of subjects from Tetschke et al. (2018) with five *in silico* parameters λ_PV_ = λ_*i*_ for each subject as detailed in Section 2.3.2.

**ID**	**γ**	**β**	***B***	***V*_pat_**	**λ_1_**	**λ_2_**	**λ_3_**	**λ_4_**	**λ_5_**
01	0.769	1.650	865.45	5530.04	0.405	0.418	0.512	0.513	0.521
02	0.388	0.867	885.42	4666.08	0.385	0.498	0.558	0.706	0.709
03	0.510	1.617	863.97	5265.93	0.326	0.393	0.413	0.480	0.549
04	0.323	0.424	854.15	5984.70	0.522	0.544	0.604	0.878	0.888
05	0.061	1.381	971.67	7734.16	0.192	0.321	0.334	0.367	0.419
06	0.590	2.615	1001.42	5096.65	0.290	0.331	0.343	0.354	0.415
07	0.262	1.518	964.59	7270.17	0.343	0.349	0.370	0.424	0.480
08	0.324	2.676	704.42	4091.19	0.216	0.243	0.340	0.371	0.433
09	0.356	0.891	958.55	9282.78	0.366	0.555	0.559	0.602	0.605
10	0.089	2.557	851.70	4588.62	0.199	0.207	0.298	0.391	0.396
11	0.243	0.925	1006.45	4610.27	0.384	0.385	0.533	0.615	0.652
12	1.003	1.409	932.51	6127.49	0.528	0.541	0.567	0.581	0.631
13	0.057	0.879	647.98	4017.69	0.198	0.369	0.428	0.506	0.583
14	0.762	0.460	1081.34	8260.98	0.639	0.743	0.767	0.787	0.845
15	0.344	2.132	939.61	6778.40	0.289	0.334	0.387	0.397	0.408
16	0.141	1.661	753.24	7102.67	0.226	0.339	0.349	0.350	0.379
17	0.470	0.544	900.53	5832.50	0.514	0.541	0.691	0.705	0.758
18	0.525	0.631	841.61	4872.18	0.529	0.661	0.689	0.695	0.847
19	0.423	1.525	786.47	5109.69	0.393	0.401	0.451	0.512	0.540
20	0.661	2.798	765.99	8486.20	0.328	0.334	0.341	0.342	0.360
21	0.686	1.943	908.60	5725.97	0.345	0.389	0.404	0.408	0.463
22	0.613	3.142	893.06	5438.46	0.278	0.303	0.305	0.337	0.342
23	0.421	1.528	695.05	4989.05	0.318	0.502	0.518	0.533	0.563
24	0.863	2.078	768.83	6182.47	0.435	0.454	0.469	0.476	0.479
25	0.414	1.172	687.85	5733.62	0.408	0.559	0.575	0.600	0.625
26	0.635	0.836	925.62	6168.52	0.659	0.681	0.682	0.708	0.746
27	0.952	1.596	869.00	6351.31	0.440	0.444	0.466	0.490	0.555
28	0.805	1.486	809.18	5987.13	0.472	0.497	0.507	0.527	0.551

*Patient-specific parameters γ and β are given, along with the B value used in that study and the calculated blood volume V_pat_*.

### 2.4. Computational Methods

In this section, the numerical methods and optimization approaches are described. First, a parameter estimation problem is solved on the available clinical data for proof-of-concept simulations. Then, optimization approaches for the generation of treatment schedules for PV patients are presented and discussed. The software used to evaluate the approach is stated in the corresponding subsection. The most relevant parts of the code are available on GitHub (https://github.com/tetschke/PVschedule).

One main focus in this paper is the generation of optimal treatment schedules for phlebotomies of PV patients. Important properties of a suitable treatment schedule include the following:

1. **Respecting an upper bound**: the principle goal of treatment is to decrease the density of RBCs in the blood (measured in *Hct*) to reduce the symptoms of the disease and to reduce the risk of fatal complications. For this purpose, with the help of physicians, an upper limit for *tHb* (*X*_3,up_) is identified, which should not be exceeded.
2. **Minimizing the number of treatments**: with a good choice of dates for when treatments will be performed, one might reduce the number of necessary treatments without violating the proposed critical thresholds. This reduces the amount of days in which the patient might have side effects because of the treatment.
3. **Incorporating restrictions of the physician**: procedures in hospitals or medical practices should be limited to regular working hours. That is, weekends and night times should not be regarded as feasible in an optimal schedule. Other restrictions of the physicians can also be incorporated into the schedule.
4. **Varying the volume of a phlebotomy**: in clinical practice, a standard amount (500 ml) of blood is typically withdrawn in a phlebotomy (Lichtman et al., 2006). This restriction can be replaced with an interval of possible volumes, which can be chosen individually for each patient.
5. **Incorporating preferences of the patient**: a patient suffering from PV usually has a normal life span and can live a normal live with all its obligations. Thus, it might be advantageous to give the patient the means to prioritize possible time slots for therapy. For instance, job-related appointments or a vacation can be included in the planning with the help of a weighted objective function.

The focus of this work lies on the first three properties. Properties 1 and 3 will be incorporated as constraints of the optimization problem. The minimization of the number of treatments is reflected in the objective function *J*. This can have the structure

(4) $$\begin{array}{l} J=\int_{0}^{T}u\left(t\right)dt \end{array}$$

in the case of a continuous problem formulation. In the integer case it is

(5) $$\begin{array}{l} J=\underset{i\in T}{\sum }U_{i}, \end{array}$$

where T is a subset of the used time discretization. A phlebotomy is a continuous process in a very short amount of time compared to the relevant time horizon for treatment planning. Therefore, the interpretation as an integer control is physiologically sensible. In contrast, the continuous objective function formulation corresponds to a minimization of the removed blood volume. Nevertheless, the latter one enables us to thoroughly analyze the structure of the resulting optimal control and yields insights into model properties. This justifies the use of these continuous solutions for the generation of integer solutions with low computational cost, as detailed in the next subsections.

For improved readability, the schedules generated by the methods presented in the following sections are abbreviated as follows:

- **H-Schedule**: Heuristic approach without constraints given by the test case (section 2.4.2).
- **HC-Schedule**: Heuristic approach with constraints given by the test case (section 2.4.2).
- **C-Schedule**: Solution of continuous optimal control problem (OCP) (section 2.4.3).
- **IP-Schedule**: Integer programming approach (section 2.4.4).
- **SUR-Schedule**: Sum up rounding (SUR) (section 2.4.5).
- **BB-Schedule**: Rounding via branch and bound (B&B) (section 2.4.6).
- **DP-Schedule**: Dynamic programming (DP) (section 2.4.7).

The number of treatments for such a schedule is then abbreviated by n_*_, where ^*^ is the one-, two-, or three-letter code of the corresponding method. For example, *n*_*H*_ describes the number of treatments according to the heuristic approach without constraints. This indexing with the respective letter code also holds for other occurring variables.

**Figure 2:**
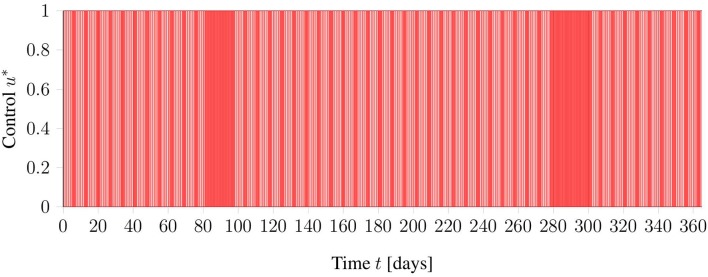
Graphical view on the general test setup including restrictions of the clinic in red. Phlebotomies are only allowed during times denoted in white.

As a general test setup for evaluating the optimization methods, a time horizon of 365 days (October 1st to September 30th) is considered. Treatments are possible from Monday to Friday, where the first of October is considered a Monday. In addition, restrictions of the clinic are included as blocked times on days 81–95 and days 280–301. The interpretation of these blocked times is that, around the winter and summer holidays, there are reduced personnel in the clinic, such that routine treatments are not performed. In Figure 2 an illustration of the restrictions can be found.

The evaluations were performed on 140 *in-silico*-generated PV patients, as described in section 2.3.2. All computations were performed on a server with 8 cores (Intel Xeon E5-2640 v3, 2.6 GHz) and 64 GB of RAM, running Ubuntu 18.04.3 LTS. Time measurements were performed using the “clock()” function from the Python package “time,” which, on Unix systems, displays the used CPU time without interruptions by other processes.

To present the following methods, it is sufficient to have a model based on ordinary differential equations, that characterizes PV. In section 3.1.1, the model *f*_PV_ is presented. For our purposes, it here suffices to state that the model includes a fraction λ_PV_ of affected progenitor cells, which influence the severity of the disease. The model dynamics have the following structure:

(6) $$\begin{array}{l} ẋ\left(t\right)=f_{\text{PV}}\left(t,x\left(t\right),\lambda_{\text{PV}},u\right). \end{array}$$

#### 2.4.1. Proof-of-Concept Simulations

To get a first impression regarding the validity of the extended model in 3.1.1, the data sets of PV patients presented in 2.3.1 were used to obtain patient specific parameter vectors *p*. This parameter vector includes the formerly relevant model parameters β and γ as well as the fraction λ_PV_ introduced by the model extension.

The following parameter estimation problem with the least-squares objective is formulated:

(7) $$\begin{array}{ll} \underset{p}{\min} & \frac{1}{2}\overset{n_{\eta }}{\underset{i=0}{\sum }}\frac{{\left(\eta_{i}-x_{3}\left(t_{i}\right)\right)}^{2}}{\sigma_{i}^{2}}+\phi \left(p\right) \end{array}$$

(8) $$\begin{array}{ll} s.t.\; & ẋ\left(t\right)=f_{\text{PV}}\left(x\left(t\right),\lambda_{\text{PV}},p,u\left(t\right)\right) \end{array}$$

(9) $$\begin{array}{l} x\left(0\right)=\left(x_{1}^{0},x_{2}^{0},\eta_{0}\right) \end{array}$$

where

- {*t*_0_ = 0, *t*_1_, …, *t*_*n*_η__} are the time points where *tHb* measurements were taken.
- η_*i*_ is the measurement value of *tHb* at time *t*_*i*_.
- *x*_3_(*t*_*i*_) is the corresponding model response at time *t*_*i*_.
- σ_*i*_ is the standard deviation of the measurement at time *t*_*i*_. As all considered data were collected by the same method under similar conditions, σ_1_ = 1 for all measurements.
- *p* is the chosen parameter vector with *n*_*p*_ entries (including $x_{1}^{0}$ and $x_{2}^{0}$).

and the regularization ϕ is selected as

(10) $$\begin{array}{l} \phi \left(p\right)=\overset{n_{p}}{\underset{i=1}{\sum }}{\left(\frac{p_{i}-p_{i}^{\text{prior}}}{p_{i}^{\text{prior}}}\right)}^{2} \end{array}$$

Here, ϕ(*p*) is a term that can be used to incorporate a priori information. In our setting, regularization to known parameter values for healthy subjects was taken from Tetschke et al. (2018). The initial base value *B* was computed as the average over all *tHb* measurements with a corresponding *Hct* value of 45% or lower. This optimization problem is solved formulated as a deterministic OCP using ampl_mintoc, a package for mixed-integer optimal control problems (MIOCP), based on AMPL (Fourer et al., 2002) and using IPOPT (3.12.10, Wächter and Biegler, 2006).

#### 2.4.2. Heuristic Approach

As displayed in 2.1.2 the aim of the treatment is to keep the patient's *Hct* level below 45%. To realize this, the standard procedure in clinical practice is the following. The *Hct* value of the patient is checked at regular intervals, selected in a fashion that ensures the critical threshold is not exceeded. As soon as the value becomes too high, a phlebotomy of constant volume takes place. Transferring this idea into algorithmic notation yields the following:

**Algorithm 1:** Heuristic approach

1: Set initial value *X*_0_ ⊳ (Initialization)
2: **for** i∈I\{0} **do**
3: $X_{i}=X_{i-1}+F\left(X_{i-1},f_{\text{PV}},\Delta_{t}\right)$ ⊳ (Integration)
4: **if** *X*_*i*, 3_>*X*_3,up_: **then** ⊳ (Check for violation)
5: **if** i∈T: **then** ⊳ (Treatment if allowed)
6: $X_{i,3}=X_{i,3}-\frac{V_{\text{max}}}{V_{\text{pat}}}\cdot X_{i,3}$
7: **else** ⊳ (Shift treatment)
8: Find largest $i^{*}\in T$ with $i^{*}+i_{\text{dwell}}<i$ and
9: Set *i* = i^*^
10: $X_{i,3}=X_{i,3}-\frac{V_{\text{max}}}{V_{\text{pat}}}\cdot X_{i,3}$

where

- I={0,…,N} is the index set corresponding to the equidistant integration grid with step size Δ*t*.
- T⊂I denotes the integration points in which a treatment is possible.
- F is the forward quadrature scheme (here, the Runge-Kutta-scheme of order 4) with regard to Model (16).
- *V*_max_ and *V*_pat_ are the constant blood volume per treatment and total blood volume of the patient, respectively.
- *i*_dwell_ is the dwell time of the system, which represents the minimal distance between two treatments.

For I=T, heuristic treatment schedules without test constraints are generated (H-Schedules). Using T as in the general test case described above, HC-Schedules are computed. One major advantage to this approach is that both types of treatment plans can be computed quickly (within a few seconds). However, the treatment plans are not guaranteed to be optimal. Moreover, this heuristic does not take the lower bound *X*_3, lo_ into account. Therefore, it is necessary to inspect other approaches.

#### 2.4.3. Continuous Optimal Control Problem

**Figure 3:**
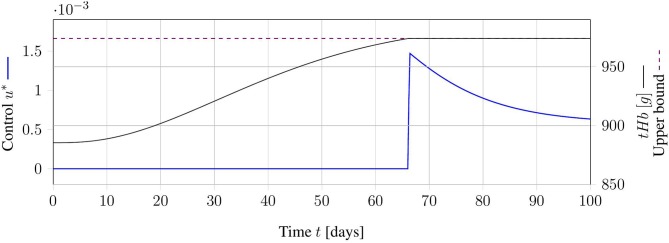
Exemplary result for an optimal relaxed treatment schedule. The continuous control function *u* (blue) is zero, as long as the *tHb*-value (black) is below the upper bound (dashed, purple). As soon as the upper bound is reached, the control function increases exactly as much as necessary to keep the *tHb*-value at the upper bound.

Another point of view is to see the desired treatment schedule as a solution to an OCP. To apply the solution in clinical practice, we are interested in a mixed integer solution. The next two sections deal with the generation of feasible and optimal integer solutions. First, we showcase the relaxed OCP and generate continuous treatment schedules (C-Schedules). An interpretation of these schedules is that a phlebotomy can be done arbitrarily often with arbitrarily withdrawn blood volumes. An exemplary illustration of a continuous solution with the corresponding *tHb* trajectory is displayed in Figure 3. Some of the rounding strategies in the following sections are based on these relaxed solutions. Further, the theoretical investigation of the solution structure can yield insights into the underlying structure of the problem.

The continuous OCP for minimizing the number of phlebotomies while allowing fractional treatments reads as follows:

(11) $$\begin{array}{l} \begin{array}{cc} \underset{u\left(.\right)}{\text{min}}\int_{0}^{T}u\left(t\right)\text{d}t\\ s.t.\;ẋ\left(t\right)=f_{\text{PV}}\left(x\left(t\right),\lambda_{\text{PV}}\right) & t\in \left[0,T\right]\\ \;x\left(0\right)=x_{0}\\ \;X_{3,\text{lo}}\leq x_{3}\left(t\right)\leq X_{3,\text{up}} & t\in \left[0,T\right]\\ \;u\left(t\right)\in \left[0,1\right] & t\in \left[0,T\right] \end{array} \end{array}$$

The objective function only indirectly accounts for the number of necessary treatments. Actually, this formulation minimizes the amount of withdrawn blood over the time horizon. A theoretical analysis of the problem solution is given in Appendix A. This analysis yields unique optimal control u^*^ of the structure:

(12) $$u^{*}(t)=\{\begin{array}{ll} 0 & ,x_{3}(t)<X_{3,\text{up}}\\ u_{\text{path}}(t) & ,x_{3}(t)=X_{3,\text{up}} \end{array}$$

This optimal control is intuitive in the sense that no treatment is applied when unnecessary. Alternatively, phlebotomies are reduced to a minimum, such that they approach the threshold *X*_3,up_. The existence of this solution shows that, in general, the OCP is solvable. Computationally, this problem is solved with a non-linear programming formulation in CasADi (3.5.1) (Andersson et al., 2019) using IPOPT (3.12.3, Wächter and Biegler, 2006).

#### 2.4.4. End Time Optimization Using Integer Approach on Non-linear Program

Continuous blood withdrawal, as seen in the case of the relaxed problem, can not be performed in clinical practice with currently available tools. To find an approach that is closer to clinical practice, an MIOCP with a discrete formulation is used. Let *U* = {*U*_1_, …, *U*_*N*_} and *X* = {*X*_1_, …, *X*_*N*_}. Then the discrete formulation is given by

(13) $$\begin{array}{l} \begin{array}{cc} \underset{U,X}{\min}\underset{i\in T}{\sum }U_{i}\\ s.t.\;X_{i+1}=\left(1-U_{i+1}\cdot \frac{V_{\text{max}}}{V_{\text{pat}}}\right)\\ \;\cdot \left(X_{i}+F\left(X_{i},f\left(X_{i}\right),\Delta t\right)\right) & \forall i\in I\backslash \left\{N\right\}\\ \;X_{3,\text{lo}}\leq X_{i+1,3}\leq X_{3,\text{up}} & \forall i\in I\backslash \left\{N\right\}\\ \;X_{0}=x_{0}\\ \;U_{i}\in \left\{0,1\right\} & \forall i\in T\\ \;U_{i}=0 & \forall i\in I\backslash T \end{array} \end{array}$$

Here, $I,T,F,V_{\text{max}}$, and *V*_pat_ are the same as in subsection 2.4.2.

Using this objective function, the system solution is not unique. In fact, a solution with $\sum U_{i}=\underset{U,X}{\min}\sum U_{i}$ does not take into account when the next treatment will take place after the end of the time horizon. A possible extension to avoid this problem is to include the time point of the next necessary treatment *T*_*f*_ after the end of the schedule. Although it is possible to combine those two objectives, it is unclear how exactly the individual components should be weighted. To circumvent this problem, an iterative approach is proposed.

Using the heuristic approach with schedule constraints T leads to a feasible treatment schedule, which gives an upper bound *u*_up_ for the necessary number of treatments. Starting with *u*_up_, we fix the number of treatments in the optimization problem and maximize *T*_*f*_. We decrement the number of treatments and repeat, until there are no more feasible solutions. The optimization problem that needs iterative solving is

(14) $$\begin{array}{l} \begin{array}{cc} \underset{U,X,X_{N+1},\ldots ,X_{N_{T}},T_{f}}{\min}-T_{f}\\ s.t.\; & X_{i+1}=\left(1-U_{i+1}\cdot \frac{V_{\text{max}}}{V_{\text{pat}}}\right)\\ \;\cdot \left(X_{k}+F\left(X_{i},f\left(X_{i}\right),\Delta t\right)\right) & \forall i\in I_{T_{f}}\backslash \left\{N_{T_{f}}\right\}\\ \;X_{3,\text{lo}}\leq X_{i+1,3}\leq X_{3,\text{up}} & \forall i\in I_{T_{f}}\backslash \left\{N_{T_{f}}\right\}\\ \;X_{0}=x_{0}\\ \;X_{N_{T}}=X_{3,\text{up}}\\ \;U_{i}\in \left\{0,1\right\} & \forall i\in T\\ \;\underset{i\in T}{\sum }U_{i}=u_{\text{sum}}\\ \;U_{i}=0 & \forall i\in I\backslash T \end{array} \end{array}$$

Here, $I_{T_{f}}=I\cup \left\{N+1,\ldots ,N_{T_{f}}\right\}$ is an expansion of the former integration index set I for additional integration points after the times where controls are possible. Thus, T⊂I is selected such that ∀i∈T:i≤N. The objective of minimizing −*T*_*f*_ reflects the aim of postponing for as long as possible the first phlebotomy after the end of the schedule with the given number of treatments. The algorithm, then, is as follows:

**Algorithm 2:** Mixed Integer OCP with *T*_*f*_

1: Solve the heuristic algorithm (1) and set *u*_up_ to objective
2: *u*_sum_ = *u*_up_
3: Solve (14)
4: **while** feasible **do**
5: *u*_sum_ = *u*_sum_−1
6: Solve (14)

This problem is solved with BONMIN (Bonami et al., 2008) using a non-linear programming formulation in CasADi. Integer schedules (IP-Schedules) derived using this MIOCP formulation have the advantage of being realizable in clinical practice while still including the main ideas for optimal treatment. However, this problem leads to an MIOCP, that is computationally expensive. In general, MIOCP problems are NP-hard. This already holds true for the linear, discretized version of this problem class (Kannan and Monma, 1978). Thus, for large |T| in particular, the problem is difficult to handle. For rather small |T|, this approach can be investigated and compared to the heuristic approach presented in subsection 2.4.2. In addition, using BONMIN on a non-linear problem does not guarantee global optimality. The performance of the software depends on the options used. In this paper, we used the following options: *variable_selection* = *most-fractional*, and *tree_search_strategy* = *dive*.

#### 2.4.5. Sum-Up Rounding

Owing to the size of the MIOCP, as described in the previous subsection, computations with standard solvers are only feasible for a rather small number of possible integer control points. Larger problem sizes might be more relevant. Indeed, more control points per week or longer overall time horizons can be included. Thus, it is worthwhile to inspect rounding strategies and to compare them to the heuristic approach.

The SUR approach (Sager, 2005) exclusively uses the optimal solution of the relaxed problem (11) to compute a binary treatment schedule. Basically, the idea is to collect the relaxed control in time and set the integer solution to one, as soon as a certain threshold *u*^*T*^ is reached. This collection is then reduced by one, and, afterwards, the previous process is repeated.

We use the multiple shooting method on an equidistant time grid for the computation of the relaxed solution u^*^. The integer solution at the discretization point *t*_*i*_ using SUR can then be computed as follows:

(15) $$U_{i}=\{\begin{array}{ll} 1 & \text{, if}\sum_{j=0}^{i}u_{j}^{*}-\sum_{j=0}^{i-1}U_{j}\geq u^{T}\\ 0 & \text{, else} \end{array}$$

In the standard SUR approach, *u*^*T*^ is set to $\frac{1}{2}$. Owing to the problem structure, we instead use *u*^*T*^ = ε for SUR-Schedules, where ε > 0 is close to zero. This is necessary because only the relaxed solution is non-zero. If the upper bound *X*_3,up_ is already reached, treatment must be done immediately.

This approach has the advantage that it is easy to implement and the computations are extremely fast, once the relaxed problem is solved. Moreover, if the relaxed problem includes blocked times *t*_*j*_, $u^{*}\left(t_{j}\right)$ will be zero and *U*_*j*_ = 0 automatically.

The big disadvantage to SUR is that it is not obvious how to include path constraints. The strategy only takes into account the relaxed solution. There is no guarantee that the upper bound *X*_3,up_ will be respected.

To summarize, although fast and intuitive to implement, SUR-Schedules risk endangering the patient, owing to violations of the treatment aim. Therefore, in clinical applications, the use of this approach should be combined with safety strategies, such as the use of a stricter upper bound *X*_3,sumup,up_ < *X*_3,up_.

#### 2.4.6. Rounding via Combinatorial Integral Approximation

Another approach to generating integer solutions from the relaxed solution is to adopt so-called combinatorial integral approximation (Sager et al., 2011). For this, we used open-source software called pycombina (Buerger et al., 2019). Here, a B&B algorithm is implemented, that is able to include combinatorial constraints with regard to binary controls. The standard B&B tree is organized in a fashion, that branches forward in time.

Originally, the algorithm was designed to approximate relaxed controls with binary ones. For this purpose, it does not need to take into account the actual states. Therefore, it is unable to deal with path constraints and suffers from the same disadvantage as the SUR approach.

This is why we adapted the algorithm to take into account the states (and especially *x*_3_) in each iteration through forward integration. If at time point *t*_*i*_ one of the conditions *X*_3,lo_ ≤ *x*_3_(*t*_*i*_) ≤ *X*_3,up_ is violated, the corresponding branch of the tree is no longer feasible and can be disregarded. This not only helps to include path constraints, but also decreases the size of the B&B tree.

This modified B&B version is able to generate feasible solutions, if we also fix the control *u* to zero when no treatments are possible. We used the prefixing option in pycombina to include this into our problem formulation.

The overall quality of BB-Schedules generated by this approach depends on the maximum number of iterations. As the B&B tree tends to become very large, relatively few iterations search through only part of that tree. This can lead to instances where no solution can be determined, however, even though we implemented the additional pruning of the tree for infeasible solutions. Nevertheless, a large number of iterations leads to very large run times. For our numerical results, we used the default of five million iterations.

#### 2.4.7. Dynamic Programming

A completely different algorithmic idea for the solution of (13) is to generate treatment schedules by dynamic programming (DP-Schedules). Here, discretization is done not only in time, but also in the state space. This approach goes back to Bellman (1957). Details can be found in Bertsekas (2012).

First, we introduce an equidistant grid $x^{0}<x^{1}<\cdots <x^{n_{x}}$ with resolution Δ_*x*_ in state space and tabulate state transitions: for each possible combination of a state value and a possible control value, the corresponding result of an integration over the next time interval must be stored. The result of the integration usually does not match one of the grid points. This is why rounding toward a valid grid point is necessary.

In our provided code this tabulation is stored with the help of indices. Thus, the rounding is done in the following fashion: Let *i* be a fractional value of a result of an integration. This value is a convex combination of the two grid points closest to the result. The value *i* is then rounded toward a valid grid point i^*^, if $-0.5\cdot \Delta_{x}\leq i-i^{*}-o\cdot \Delta_{x}\leq 0.5\cdot \Delta_{x}$ holds. For the offset *o* = 0.0, rounding half up is applied, whereas for *o* > 0, a more conservative rounding is applied. We test both *o* = 0.0 and *o* = 0.4.

The tabulation is then used to compute a so-called cost-to-go function. For each time point and state grid point this function indicates the best possible choice from that state and that time onwards. This is computed backwards in time. The last step is the computation of the optimal control starting in suitable grid points close to *x*_0_ with the help of the tabulation.

This approach is globally optimal with regard to the grid used, as every possible combination of states and controls is evaluated. However, this approach suffers from practical drawbacks, when systems with many states are used, or when there are too many grid points for each state. In the case of the MIOCP (13), only three states have to be regarded and we consider only binary control. For this reason, the algorithm might be a good choice. We used 400 grid points for each of the three states.

After the initial tabulation, the algorithm has a linear complexity in the time discretization. Therefore, this approach is especially suited for schedule generation with large time horizons. It is also easy to include constraints. In our implementation, we worked with sparse matrix structures to account for the exponential growth of the state transition tabulation.

## 3. Results

In this section, the results based on the previously introduced methods are presented. The model proposed by Tetschke et al. (2018) is extended, and we discuss necessary assumptions for the biological process. The plausibility of this extended model is then examined with both steady-state analysis and numerical proof-of-concept simulations using clinical data from PV patients. Then, the numerical results from heuristic generations of treatment schedules are compared to those of other numerical approaches on *in silico* patient configurations.

### 3.1. Mathematical Modeling of Erythropoiesis in PV Patients

The three-compartment model by Tetschke et al. (2018) captures the basic physiological processes of healthy erythropoiesis in adults. We extend this model to capture PV as well. The small number of free parameters in the original model also motivated its suitability for this purpose: the amount of clinical data describing PV patients is usually insufficient for large models.

In this section, we describe the proposed model for PV, analyze its properties, and discuss simulation results using clinical data. We generated suitable treatment plans using heuristic and optimization-based approaches. The overall goal of treatment was to ensure the safety of the patient, while aiming to improve quality of life.

#### 3.1.1. Model Extension

Here, we discuss our extension of the model (1) to reflect the relevant dynamics of erythropoiesis in PV patients. For this, we follow the idea in Eaves and Eaves (1978) stated in subsection 2.1.1. According to this study, a fraction of CFU-E cells proliferates at a maximal rate, independent of EPO or fractional blood loss. We introduce a parameter λ_PV_, which corresponds to this fraction and can take values between [0, 1]. Correspondingly, there is a fraction of cells 1−λ_PV_ that responds in a normal way to EPO. A person not affected by PV will correspond to λ_PV_ = 0, whereas higher values give means to quantify the severeness of the disease. As the compartment *x*_1_ mainly consists of CFU-E cells, an intuitive model extension of (1) is given as follows:

(16) $$\begin{array}{l} \;{ẋ}_{1}=\beta \cdot \left(X_{0}-k_{1}\cdot x_{1}\right)+\left(1-\lambda_{\text{PV}}\right)\cdot Fb\left(x_{3}\right)\cdot x_{1}\\ \;+\lambda_{\text{PV}}\cdot \gamma^{*}\cdot x_{1}\\ \begin{array}{c} \;{ẋ}_{2}=\beta \cdot \left(k_{1}\cdot x_{1}-k_{2}\cdot x_{2}\right) \end{array}\\ \begin{array}{c} \;{ẋ}_{3}=\beta \cdot \left(k_{2}\cdot x_{2}-\alpha \cdot x_{3}\right)\\ \;Fb\left(x_{3}\right)=\gamma \cdot \left(1-\frac{x_{3}}{B}\right) \end{array} \end{array}$$

with γ^*^ denoting the growth rate of affected cells in *x*_1_. A phlebotomy can be incorporated in the same way as Equation (2) in section 2.2.

The model components are here discussed with respect to their plausibility in the case of PV.

- β, *k*_1_, *k*_2_, γ: using this model extension by cell partition with λ_PV_ leads to the assumption that cells affected by PV only proliferate faster in *x*_1_, and otherwise behave like a healthy cell. We note that there might be physiological processes not covered by this model that affect other components, such as the transition times between the compartments. However, we assume that this is not the case and use the model variables β, *k*_1_, *k*_2_, and γ as in Tetschke et al. (2018).
- α: there are conflicting studies regarding the average life span of erythrocytes in PV patients. Depending on the investigation, the average life span is either shortened or normal (see London et al., 1949; Huff et al., 1950; Berlin et al., 1951). We will not discuss this further here. We used $\alpha =\frac{1}{120}$ as in the healthy case. We note that α might be different in PV patients and might depend, for example, on progression of the disease, reflected by λ_PV_, or on patient-specific factors. This could be inspected in a follow-up investigation, once suitable clinical data are available.
- γ^*^: the model variable γ^*^ has a significant impact on proliferation in PV patients, especially in those with a higher number of affected cells described by high values of λ_PV_. To our knowledge, however, no study has investigated the proliferation rate of CFU-E in PV patients based on the fraction of affected cells. Therefore, an accurate guess for the value of γ^*^ is not possible. In case of unknown model variables, a numerical estimation based on suitable data is optimal. However, there are many unknown patient-specific variables, such as β, γ, λ_PV_, and (in most cases) *B*. The additional estimation of γ^*^ is unreasonable, given that data of exceptional quality and quantity are unavailable. As the available data do not often meet these criteria, one might opt for a heuristic approach by assuming a dependency of γ^*^ on other model variables, such as γ or β. By definition, γ reflects a proliferation amplification of EPO-affected cells, such that the use of the EPO-independent factor β seems more intuitive. For our investigation, we used $\gamma^{*}=\frac{\beta }{10}$.
- *X*_0_: the model variable *X*_0_ reflects the inflow from hematopoietic stem cells to the erythrocyte lineage. As the proliferation rate of PV-affected stem cells might also be increased, one might assume *X*_0_ to be higher and to be dependent on λ_PV_. We assumed that a potentially enhanced stem cell inflow is compensated by the proliferation rate γ^*^, and we used *X*_0_ as in Tetschke et al. (2018).

#### 3.1.2. Steady State Analysis

In most cases, the system's steady state for the erythrocyte mass ${\overset{-}{x}}_{3}=B_{\text{PV}}$ of PV patients should be at sufficiently high levels such that long, before it is reached, treatment is administered to prevent possibly fatal complications. However, deriving information about the system's steady states often yields useful information about the system's properties. In this case, we inspected the relation between the new steady state erythrocyte mass *B*_PV_ and the steady state erythrocyte mass *B* without the model extension.

Following the calculations in Appendix B, the steady state erythrocyte mass *B*_PV_ is given by

(17) $$B_{\text{PV}}(\lambda_{\text{PV}})=B\cdot \{\begin{array}{c} -\frac{\beta \cdot k_{1}-(1-\lambda_{\text{PV}})\cdot \gamma -\lambda_{\text{PV}}\cdot \gamma^{*}}{2\cdot (1-\lambda_{\text{PV}})\gamma }\text{ for }\lambda_{\text{PV}}<1\\ +\sqrt{{\left(\frac{\beta \cdot k_{1}-(1-\lambda_{\text{PV}})\cdot \gamma -\lambda_{\text{PV}}\cdot \gamma^{*}}{2\cdot (1-\lambda_{\text{PV}})\gamma }\right)}^{2}\;+\;\frac{\beta \cdot k_{1}}{(1-\lambda_{\text{PV}})\cdot \gamma }},\\ \frac{\beta \cdot k_{1}}{\beta \cdot k_{1}-\gamma^{*}},\text{ for }\lambda_{\text{PV}}=1 \end{array}$$

As described in the Appendix, we also found that this function (using $\gamma^{*}=\frac{\beta }{10}$) is continuous for λ_PV_ ∈ [0, 1], such that only the case where λ_PV_ = 1 must be thoroughly investigated. With similar calculations, one can also show that *B*_PV_(λ_PV_) increases monotonously for λ_PV_ ∈ [0, 1].

To summarize the results, *B*_PV_ is a continuous, monotonously increasing function with *B*_PV_(λ_PV_) ∈ [*B*, 5·*B*] for λ_PV_ ∈ [0, 1]. This means that an increasing fraction of affected cells can indeed lead to physiological complications, as the system tends to reach critical erythrocyte levels. This is consistent with the main physiological assumptions about the process.

### 3.2. Numerical Results

In this section, the numerical results using the proposed model are presented. First, clinical data are evaluated in a proof-of-concept simulation. Then, the computed treatment schedule given by the heuristic method in section 2.4.2 is compared to schedules computed by the other approaches given in 2.4.

In 22 of the available 140 test cases, no H-Schedules could be generated, owing to the constraints. The remaining 118 H-Schedules were thus compared to the schedules from other methods.

#### 3.2.1. Proof of Concept Simulation

**Figure 4:**
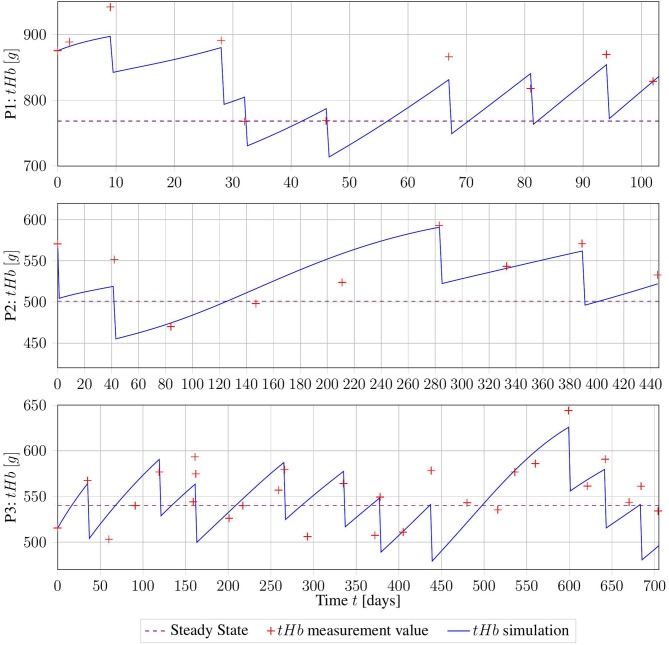
Erythrocyte trajectories as a result of parameter estimation on three clinical data sets. The computed measurement values are given in red, and the healthy base value *B* is displayed in purple.

The three data sets of patients suffering from PV presented in section 2.3.1 were used to assess the applicability of the model to real-world data. The method described in section 2.4.1 was used to obtain the patient-specific parameter vector *p* = (β, γ, λ_PV_). The results are displayed in Figure 4 and summarized in Table 3.

**Table 3:** Results of proof-of-concept simulation of clinical data from three patients.

**Patient ID**	**β**	**γ**	**λ_PV_**	***B***	***B*^*PV*^**	***R*^2^**
01	0.6	0.3	0.89	768.25	1314.20	0.89
02	0.2	0.1	0.62	501.04	607.02	0.76
03	0.2	0.1	0.61	540.17	650.21	0.45

Taking into account all the problems with the collected data, the fits of the trajectories appear satisfying from visual inspection. Objectively, the *R*^2^ value of the three fits was 0.7. However, for subjects 02 and 03, the parameters β and γ were both equal to the lower bound set, owing to numerical restrictions. This might be a sign of errors in the assumption of *B*, or in the calculation of *tHb* values from *Hct*. More precise information about those factors will drastically improve the numerical performance of the method.

The good fit achieved by this method suggests that our proposed model captures the essential dynamics of this process. However, this must be verified using higher-quality clinical data.

#### 3.2.2. Evaluation of Integer Approach

In this section, we compare the HC-Schedules and the IP-Schedules of the MIOCP approach in Algorithm 2. For demonstration purposes, the IP-Schedule was compared to the corresponding H-Schedule and HC-Schedule in one modified test case. For this test case, subject 20 with λ_PV_ = λ_2_ was used with a time horizon of *T* = 103 days. Per allowed day, one time point for treatment was possible. Four sets of test restrictions on weekdays were tested: treatments were exclusively allowed on Monday (Mo), Monday and Wednesday (Mo, Wed), Monday, Wednesday, and Friday (Mo, Wed, Fri), or Monday, Wednesday, Friday, and Sunday (Mo, Wed, Fri, Sun)—beginning the simulation with the first day being a Monday. An integrator step size of $\frac{1}{6}$ days was used. The results are displayed in Table 4.

**Table 4:** Results of integer approach run time demonstration.

**Days per week**	***n*_H_**	***n*_HC_**	***n*_IP_**	**Δ_Tf_[*d*]**	**CPU time MIOCP (s)**
1	4	5	5	0	954.8
2	4	5	5	0	17960.4
3	4	5	5	0	72239.5
4	4	5	5	0	195409.4

Here, Δ_Tf_[*d*] = *Tf*_HC_ − *Tf*_IP_, where both *Tf*_HC_ and *Tf*_IP_ are computed as the respective time points in which the first treatment after the observed time horizon occurs. In the documentation, we set Δ_Tf_: = 0 when $|\Delta_{\text{Tf}}|<\frac{1}{6}$. The interpretation is that a time deviation below this step size is irrelevant, and small numerical differences should not be incorporated.

In this test case, the results of the IP-Schedules and HC-Schedules were without notable differences. However, whereas the generation of HC-Schedules had a constant run time of only a few seconds, the run time of IP-Schedules dramatically increased (up to 2.3 days for the largest test case). This demonstrates that the MIOCP approach is only suitable for very small test cases. Therefore, applications for this approach to the general test case in subsection 2.3.2 are unfeasible.

To compare the heuristic approach with the MIOCP approach further, both algorithms were applied to a modified version of the test case. It was modified with a smaller end time *T* = 103 permitting treatments only on 1 day per week (Mo) and only at one time point per day.

In three cases, the MIOCP approach did not produce a feasible solution. In all other cases, the number of treatments *n*_IP_ and *n*_HC_ were equal. In those cases, differences only occurred with different Δ_Tf_. In two of the latter cases, the MIOCP schedules were worse by $\bar{\left|\Delta_{\text{Tf}}\right|}=3.62\pm 0.911$ days. In five other treatment schedules, the heuristic solution produced better results by $\bar{\left|\Delta_{\text{Tf}}\right|}=0.402\pm 0.1$. Another six subjects were excluded, as no treatment was necessary owing to the shortened time horizon. In the other 124 cases, no significant differences between the two approaches were found.

**Figure 5:**
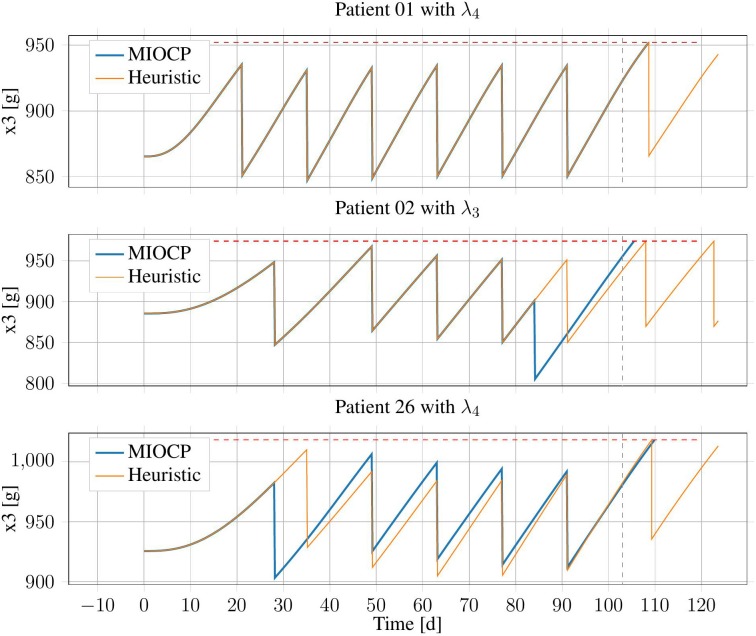
Erythrocyte trajectories of three exemplary patients using IP-Schedules and HC-Schedules. The upper threshold (red, dashed) and the end of the time horizon at *T* = 103 days (gray, dashed) are marked.

Exemplary results from three patient configurations are displayed in Figure 5. Patient 01 with λ_PV_ = 0.51 is an example of the general case, in which both generated treatment schedules were identical. By contrast, for patient 02 with λ_PV_ = 0.56, the IP-Schedule was worse, owing to a treatment at approximately *t* = 84 days. As solutions generated using BONMIN can be especially sensitive to the algorithmic options, this results could likely be improved by testing more configurations. There are also examples where the IP-Schedule was slightly better, such as the case of patient 26 with λ_PV_ = 0.71.

The MIOCP optimization approach using BONMIN only rarely yielded an improvement over the heuristic approach. The original problem size (see subsection 2.3.2) had to be reduced by a factor of 17 in terms of the number of integer variables, to produce results in a reasonable amount of time. Nonetheless, the run-times were long (920.22 ± 845.71 s). Therefore, the use of standard integer optimization solvers seems inappropriate for this problem. This motivated the investigation of other heuristic approaches, such as rounding schemes, for generating treatment plans.

#### 3.2.3. Sum-Up Rounding

In this section, the HC-Schedules and the SUR-Schedules are compared. One relevant property is the difference in the number of treatments *n*_diff_ = *n*_HC_ − *n*_SUR_ of the schedules. The sum-up method does not directly take into account the critical threshold *X*_3,up_. Therefore, we evaluated the number of days in which the threshold was violated (*d*_viol_).

In all 140 test cases, SUR-Schedules were successfully computed. In 118 cases where the heuristic also found a feasible solution, the sum-up approach on average had a lower objective function value than the respective HC-Schedules, by an average of ${\overset{-}{n}}_{\text{diff}}=1.15\pm 3.92$ treatments. However, using these 118 treatment SUR-Schedules, the patients *tHb* was above the critical level for ${\overset{-}{d}}_{\text{viol}}=58.93\pm 70.81$ days of that year. This was also the case for the 22 SUR-Schedules, with which the heuristic method did not find a valid solution (${\overset{-}{d}}_{\text{viol}}=74.53\pm 38.4$). There was no case in which the SUR-Schedule was better (by having fewer treatments or being the only approach that worked), with zero days of violation.

**Figure 6:**
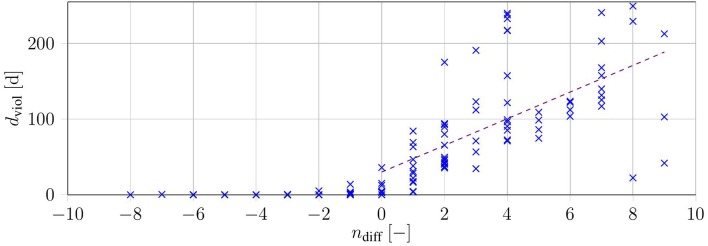
Duration of constraint violation *d*_viol_ over the difference in the number of treatments for each of the 140 test cases (blue). The purple dashed line shows a linear regression over all instances with *n*_diff_ ≥ 0.

We investigated the reduction in treatment *n*_diff_ by the sum-up method and plotted it over the respective days of violation *d*_viol_ (see Figure 6). The data show that violations by the method increased with further reduction in the number of treatments. This was emphasized by a linear regression with a positive slope (*d*_viol,reg_(*n*_diff_) = 17.61·*n*_diff_ + 30.04[*days*] with *R*^2^ = 0.42). The regression only considered the instances with a lower objective function value in the SUR-Schedules.

In summary, the SUR-Schedules either had fewer treatments than the respective HC-Schedules, with considerable endangerment to the patient, or were similar or worse than schedules with only slight endangerment in most cases. To overcome these constraint violations, we can decrease the critical threshold *X*_3, up_, although this would lead to more treatments. Based on our investigation, the sum-up method performed considerably worse, because it did not directly take the upper bound into account.

#### 3.2.4. Rounding via Branch and Bound

The BB-Schedule was considered as a rounding approach. In contrast to the SUR-Schedule, the BB-Schedule respects constraints. As a complete B&B tree grows exponentially in the number of variables, the computations were run with a maximal number of iterations. In Table 5 we present the default results of pycombina (5 million iterations) and results from decreasing that number to half a million iterations, which increased the speed of the computations by a factor of nearly 10, omitting the time for the solution of the C-Schedule (on average 26.48 s).

**Table 5:** Results of BB-Schedule in comparison to HC-Schedule.

**Method**	**# Successful**	${\bar{n}}_{*}$	**Average CPU time (s)**
HC	118	15.78 ± 7.34	7.3 ± 13.3
BB small	111	15.47 ± 7.21	26.9 ± 3.54
BB large	112	15.54 ± 7.21	7.3 ± 13.3

In comparison to the HC-Schedule, the results of the approach are similar: 22 cases were not feasible with either approach. Additionally, the BB-Schedule failed to find a feasible solution with six patients in the version with a large iteration number (and with seven patients in the faster version). In both cases, there were 13 cases where the heuristic saved one phlebotomy, and two cases where even two phlebotomies were saved in comparison to the BB-schedule.

The results for the BB-Schedule can be improved by increasing the permitted number of iterations even further, although this would increase the average computation time.

#### 3.2.5. Dynamic Programming

The DP approach generates treatment schedules by exploring all possible schedules on a chosen grid. Those DP-Schedules were compared to the corresponding HC-Schedules. Relevant properties were the difference in the number of treatments *n*_diff,0_ = *n*_HC_ − *n*_DP,0_ and *n*_diff,0.4_ = *n*_HC_ − *n*_DP,0.4_, and the number of failed attempts for both rounding offsets. Moreover, the computation time and the used RAM were documented. The latter was the limiting factor of the approach.

**Figure 7:**
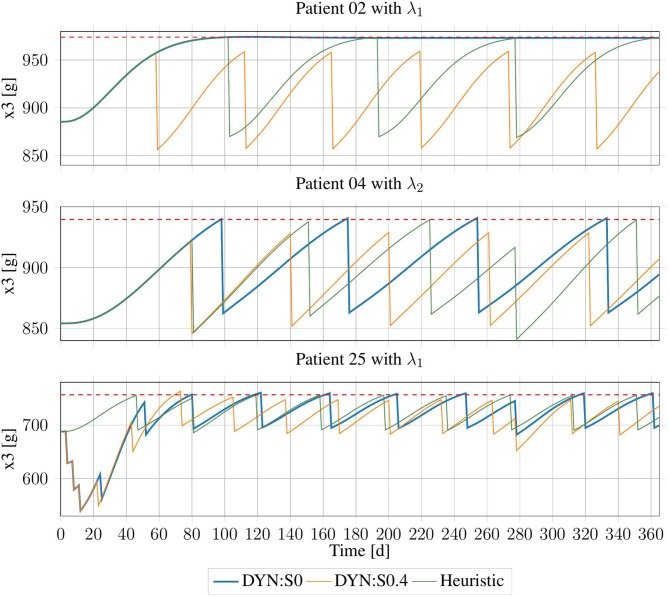
Erythrocyte trajectories using DP-Schedules for both rounding approaches and HC-Schedules for three exemplary patients. *X*_3,up_ is shown as the red dashed line.

Of all 140 patient data sets, the system memory was exceeded in four configurations of subject 08 (λ_1_, λ_2_, λ_4_, and λ_5_) for both offsets. Therefore, only the results for the other 136 data sets were available. The system memory per configuration in most cases was close to the maximum available memory (~50 GB). The results are presented in Figure 7.

Using the conservative rounding rule with offset *o* = 0.4, in an additional 12 cases, no DP-Schedule could be produced. The remaining 124 schedules on average were worse than the heuristic schedules by ${\overset{-}{n}}_{\text{diff},0.4}=-3.06\pm 1.71$ treatments, with an average violation of ${\overset{-}{d}}_{\text{viol}}=0.2\pm 0.89$ days. There was no case in which a DP-Schedule needed fewer treatments than its respective HC-Schedule.

For the commercial rounding rule with *o* = 0.0, in five data sets, no feasible solution was produced by the DP method. However, this approach was successful in four cases, in which no HC-Schedule could be generated. For the 126 cases in which both approaches succeeded, an average improvement of the DP-Schedules by ${\overset{-}{n}}_{\text{diff},0.0}=0.076\pm 1.69$ treatments was achieved with an average cost of ${\overset{-}{d}}_{\text{viol}}=9.09\pm 8.52$ days of violation. For the four cases in which the heuristic rule did not produce a schedule, the DP method had an average violation of ${\overset{-}{d}}_{\text{viol}}\;=\;5.08\;\pm \;1.17$ days.

There was no case in which an improvement from the DP method had zero days of violation. However, in some cases, DP-Schedules with only minor violations and a significant reduction in the number of treatments were generated. For example, for subject 02 with λ_1_, there was a violation of *d*_viol_ = 32.33 days with very small offset from the upper bound, which then reached its *B*_PV_, slightly below that threshold. As that threshold would probably be selected with some safety region, this subject might not need any treatment at all. Following the HC-Schedule, three treatments were applied, because the upper bound must be strictly respected. A similar case was inspected for subject 04 with λ_2_, where the number of treatments was reduced by one when *d*_viol_ = 5.5 days of violation were tolerated. There were also some cases in which the DP-Schedules were clearly sub-optimal (see subject 25 with λ_1_).

Using DP with *o* = 0.4 often produced solutions, which were feasible, but on average significantly worse than the heuristic schedules. Using commercial rounding with *o* = 0.0 provided the opportunity of finding better schedules, which only slightly endangered the patient but increased the quality of life of the patient. Therefore, this approach seems suitable for producing alternative treatment schedules, which, in clinical practice, can be compared to one another.

## 4. Discussion

### 4.1. Model

To our knowledge, this is the first time that erythropoiesis in PV patients has been described in a framework that can simulate the dynamics of the disease. This is a first step toward clinical decision support, with which medical doctors can use simulation results to predict follow-up treatments. Such a framework has the potential to improve the treatment of PV patients significantly, while decreasing the work-load of clinical personnel by reducing the number of necessary appointments.

There are some drawbacks to the proposed model, however, and these will be addressed in future work. First, the different stages of erythropoiesis are simplified and summarized in few compartments. One can argue that too much information is lost through the agglomeration of complex underlying phenomena.

Second, further investigation in this area is limited by the data available. As PV is a rather rare disease, data sets are difficult to come by. In addition, clinical measurements are performed using *Hct*, rather than with more precise values, such as *tHb*. The inclusion of *tHb* measurements in the clinical routine would drastically improve the results provided by a modeling framework, as discussed in Tetschke et al. (2018). Overall, the use of more patient data with higher density and more precise measurement techniques is necessary for the success of model-based decision support.

Finally, PV is not yet fully understood. This makes the modeling process difficult, as more black-box components must be introduced. However, the modeling framework can support medical research in this field. For example, investigations are warranted regarding the shortened life span of RBCs which often occurs in PV patients, and regarding the connection between the fraction of affected cells λ_PV_ and the JAK2V617F allele burden. Additional medical parameters might be introduced into this model framework for this purpose, which can, in combination with more clinical data, lead to new insights into the disease.

### 4.2. Optimization

In the second part of this paper, we evaluated different methods of generating treatment schedules for PV patients based on the proposed model. An overview over the results is given in Table 6.

**Table 6:** Summary of relevant properties of the investigated methods for generating treatment schedules.

**Schedule**	**H**	**HC**	**C**	**IP**	**SUR**	**BB**	**DP (0.0/0.4)**
Integer solution	✓	✓	x	✓	✓	✓	✓
No constraint violation	✓	✓	✓	✓	x	✓	x
Respecting restrictions of clinic	x	✓	✓	✓	✓	✓	✓
Extension for weighted dates	x	x	✓	✓	✓	(✓)	(✓)
Run time practicable	✓	✓	✓	x	✓	(✓)	(✓)
Memory practicable	✓	✓	✓	✓	✓	✓	x
# Computed schedules (of 140)	140	118	140	0	140	112	136/136
# Feasible instances (of 140)	140	118	140	0	33	112	8/94

*Fields with a ✓show that the respective method fulfills this property. In cases of a ✓in brackets, the method has this feature formally but with practical drawbacks. Unfulfilled properties are marked with an “x”*.

The heuristic method of generating schedules follows the intuitive treatment design practiced by medical doctors. The resulting H-Schedules and HC-Schedules can be derived quickly and the schedules are integer solutions by design. Unfortunately, the heuristic is less flexible with regard to the inclusion of new features. As this method was sub-optimal in a formal sense, the quality of this approach was evaluated in comparison to formally derived optimization methods.

The investigated methods led to treatment schedules that in most cases had an equal or higher number of treatments in the observed time horizon, or included violations of safety constraints. Both the I-Schedules and the BB-Schedules were often similar to the respective HC-Schedules. The BB-Schedules were in a few cases even slightly better than the HC-Schedules. However, those approaches are difficult to realize, owing to high run times. The generation of I-Schedules is only possible for very limited time horizons and reduced treatment options. BB-Schedules can be generated relatively quickly, but need a higher run time for an increased rate of successful computation.

It is crucial to respect safety constraints to prevent endangering patients. Therefore, the SUR-Schedules and the DP-Schedules, which do not respect these safety constraints, must be used carefully. The SUR-Schedules were in most cases worse than the corresponding HC-Schedules, and often had significant violations of the constraints. Any strategy that uses this approach will require tighter safety constraints. Consequently, this might lead to feasible treatment schedules, but they would be significantly worse than the HC-Schedules. Therefore, the sum-up approach is not recommended for generating treatment schedules. By contrast, DP-Schedules in many cases demonstrated comparable quality, without any or with only minor constraint violations. There were even cases in which the acceptance of a minor violation led to considerably improved treatment plans. The major drawback here is that the order of magnitude of the violations depends on the selected discretization. This considerably influences memory consumption. Although DP-Schedules can be used in conjunction with the corresponding HC-Schedules, the high demand for system memory renders the approach difficult to realize.

Based on our investigation using a test configuration, the heuristic method with its HC-Schedules seemed to be the method of choice for generating treatment schedules. However, the heuristic method is difficult to extend when the properties of the treatments change. For example, as a quality-of-life feature for the patient, day-based weights might be introduced, assigning more weight to inconvenient days that are preferably avoided. This would give the patient the opportunity to realize treatment on more convenient days—offering more flexibility than a strictly optimal treatment schedule. The patient can thus avoid appointments that conflict with personal commitments. Such day-based weights can be incorporated into all of the other investigated methods. This would make BB-Schedules, DP-Schedules, and (in smaller instances) IP-Schedules desirable suggestions for patient treatment. In all cases, treatment schedules can be used to support decision-making by medical doctors when planning therapy.

Continuous treatment schedules were briefly discussed, but only as a foundation for other approaches, such as the sum-up method and the B&B method. Currently, continuous phlebotomy is technologically impractical in clinics, which makes C-Schedules inapplicable. With increasing technological progress, however, such a method might be derived in the future. Based on the results of this paper, this would lead to superior treatment compared to discrete approaches.

### 4.3. Conclusion

In this paper, a novel compartment model for PV patients was derived from the data-driven model in Tetschke et al. (2018). Theoretical model analysis and proof-of-concept simulations on clinical data emphasize that this model delivers a plausible description of changes in erythropoiesis from PV.

This gives the opportunity to simulate the disease patient individually and to provide phlebotomy schedules based on this information. Due to the model structure this can be achieved using tools of mathematical optimization. Thus, in the future many different further aspects of the clinical practice can be included in the treatment design. For example, also a treatment with chemotherapy could be included into the model to also capture more severe cases of the disease. This is a first step toward clinical decision support in the case of the disease PV.

## Data Availability Statement

All datasets generated for this study are included in the article/Supplementary Material.

## Ethics Statement

The studies involving human participants were reviewed and approved by Ethics Committee of the Otto von-Guericke University at the Medical Faculty and at the University Hospital Magdeburg. The patients/participants provided their written informed consent to participate in this study.

## Author Contributions

PL and MT developed the model, performed the analysis and experiments, and wrote the paper. TF and ES contributed to the design of the research, provided clinical data for the experiments, and analyzed the data. SS contributed to the design of the research and to the discussion of the mathematical model.

## Conflict of Interest

The authors declare that the research was conducted in the absence of any commercial or financial relationships that could be construed as a potential conflict of interest.

## Abbreviations

B&B: branch and bound
BFU-E: blast forming unit-hematopoietic
CFU-E: colony forming unit-hematopoietic
DP: dynamic programming
EPO: erythropoietin
Hct: hematocrit
MCH: mean corpuscular hemoglobin
MIOCP: mixed-integer optimal control problem
OCP: optimal control problem
PV: polycythemia vera
RBC: red blood cell
SUR: sum up rounding
tHb: total hemoglobin mass.
