# Supplementary material for: Optimized and Personalized Phlebotomy Schedules for Patients Suffering From Polycythemia Vera
Source: Front Physiol. 2020 Apr 17;11:328. doi: 10.3389/fphys.2020.00328 (PMC7180210; doi:10.3389/fphys.2020.00328)
Supplement: Supplementary file 1 [file Presentation_1.zip › appendix_files/appendix_only.pdf]

APPENDIX

A Formulation as a continuous optimal control problem

Here, we describe the detailed calculations of the control trajectory u^* from Section 2.4.3. We used Pontryagins maximum principle on the continuous problem formulation to derive the properties of the optimal control trajectory. The occurring Lagrange multiplier, which in standard notation is as λ, is renamed to l to prevent confusion with the variable λ_{PV}. For further details on this procedure, we refer the reader to Bryson and Ho (1975).

In the continuous OCP, we try to minimize the donation volume over the time horizon $[0, T]$. Equation (16) with continuous phlebotomy by Equation (2) and according to constraints can be rewritten as:

$$\begin{aligned}
&\min_{u(\cdot)} && \int_0^T u(t)\mathrm{d}t \\
&\text{subject to} && \\
&\dot{x_1}(t) && = \beta\left(X_0 - k_1 \cdot x_1(t)\right) + \gamma \cdot (1-\lambda_{\text{PV}}) \cdot \left(1 - \tfrac{x_3(t)}{B}\right) \cdot x_1(t) + \lambda_{\text{PV}} \cdot \gamma^* \cdot x_1(t) \\
&\dot{x_2}(t) && = \beta\left(k_1 \cdot x_1(t) - k_2 \cdot x_2(t)\right) \\
&\dot{x_3}(t) && = \beta\left(k_2 \cdot x_2(t) - \alpha \cdot x_3(t)\right) - u(t) \cdot \tfrac{V_{\text{max}}}{V_{\text{pat}}} \cdot x_3(t) \\
&x(0) && = x_0 \\
&c_{\text{up}}(t) && = X_{3,\text{up}} - x_3(t) \geq 0 \\
&c_{\text{lo}}(t) && = x_3(t) - X_{3,lo} \geq 0 \\
&u(t) && \in [0, 1]
\end{aligned} \tag{A1}$$

The control function $u(\cdot)$ occurs linearly in the right-hand side of the system f_{PV}. Therefore, we try to find the switching function of the Hamiltonian, given by

$$\mathcal{H} = -\mathcal{L} + l^T \cdot f_{\text{PV}} - \mu \cdot c^{(q)} \tag{A2}$$

with the Lagrange term $\mathcal{L} = u$ and Lagrange multipliers l. Here, $\mu \leq 0$ with $\mu \neq 0$ if the respective constraint c is violated. Further, q is the minimal number of time derivatives of $c_{\cdot}(\cdot)$ until the expression is directly dependent on u. For both constraint equations $q = 1$ as

$$\begin{aligned}
c_{\text{up}}^{(1)} &= -f_{\text{PV},3}(t) \\
c_{\text{lo}}^{(1)} &= f_{\text{PV},3}(t)
\end{aligned}$$

and $f_{\text{PV},3}$ directly depends on u, as can be seen above. We now try to find a reformulation of the Hamiltonian $\mathcal{H}$ in the form of

$$\mathcal{H} = u(t) \cdot S(\cdot) + \text{expressions independent of u} \tag{A3}$$

From Equation (A2) it follows that

$$\begin{aligned}
\mathcal{H} &= -\mathcal{L} + l^T \cdot f - \mu \cdot c^{(1)} \\
&= -u(t) + l_1 \cdot f_{\text{PV},1} + l_2 \cdot f_{\text{PV},2} + (l_3 \pm \mu) \cdot f_{\text{PV},3} \\
&= u(t) \cdot \left[-1 - (l_3 \pm \mu) \cdot \frac{V_{\text{max}}}{V_{\text{pat}}} \cdot x_3(t)\right] + l_1 \cdot f_{\text{PV},1} \\
&\quad + l_2 \cdot f_{\text{PV},2} + (l_3 \pm \mu) \cdot \beta \cdot (k_2 \cdot x_2(t) - \alpha \cdot x_3(t)) \\
\Rightarrow S(x, l, \mu) &= -1 - (l \pm \mu) \cdot \frac{V_{\text{max}}}{V_{\text{pat}}} \cdot x_3(t)
\end{aligned}$$

where $+\mu$ if c_{up} is active, and $-\mu$ if c_{lo} is active.
We first investigate the case where $\mu = 0$ on an interval $\left[0, t_f\right]$, i.e., where no constraint is active. To evaluate the switching function S on this interval, we look at the adjoint equations and the adjoint final value for λ:

$$\begin{aligned}
-\dot{l}_1 &= \mathcal{H}_{x_1} = \beta \cdot k_1 \cdot (l_2 - l_1) + l_1 \cdot \gamma \cdot (1 - \lambda_{\text{PV}}) \cdot \left(1 - \frac{x_3}{B}\right) + l_1 \cdot \lambda_{\text{PV}} \cdot \gamma^* \\
-\dot{l}_2 &= \mathcal{H}_{x_2} = \beta \cdot k_2 \cdot (l_3 - l_2) \\
-\dot{l}_3 &= \mathcal{H}_{x_3} = -l_1 \cdot \frac{\gamma \cdot \lambda_{\text{PV}}}{B} \cdot x_1 - l_3 \cdot \left(\alpha - u(t) \cdot \frac{V_{\text{max}}}{V_{\text{pat}}}\right) \\
l(t_f) &= \frac{\partial E}{\partial x}(x(t_f))^T = 0
\end{aligned}$$

The substitution $t \to t_f - t$ yields the equivalent initial value problem on $\left[0, t_f\right]$:

$$\begin{aligned}
\dot{l} &= \mathcal{H}_x \\
l(0) &= 0
\end{aligned}$$

As the initial value of the system is zero and all expressions on the right-hand side depend on components of l as a factor, the solution to this IVP on this interval is $l \equiv 0$. Therefore, $S(x, l) \equiv -1 < 0$ and $u^* = u_{\text{min}} = 0$ on the interval $\left[0, t_f\right]$. Thus, no control will be active if none of the constraints are violated.

Starting at the former steady-state value of the system for $\lambda_{\text{PV}} > 0$, the number of erythrocytes x_3 will grow until the new steady state B_{PV} is reached or the constraint c_{up} becomes active. If B_{PV} is reached before c_{up}, the system will not change without further external influence, and $u = 0$. If instead, c_{up} becomes active, both $c_{\text{up}}^{(0)} = 0$ and $c_{\text{up}}^{(1)} = 0$ hold. From the latter equation, we derive a path control u_{path}:

$$
\begin{aligned}
0 = c_{\text{up}}^{(1)} &= f_{\text{PV},3} \\
&= \beta \left(k_2 \cdot x_2(t) - \alpha \cdot X_{3,\text{up}}\right) - u_{\text{path}} \cdot \frac{V_{\max}}{V_{\text{pat}}} \cdot X_{3,\text{up}} \\
\Leftrightarrow u_{\text{path}} &= \frac{\beta \cdot \left(k_2 \cdot x_2(t) - \alpha \cdot X_{3,\text{up}}\right)}{\frac{V_{\max}}{V_{\text{pat}}} \cdot X_{3,\text{up}}}
\end{aligned}
$$

As the path control is designed such that $f_{\text{PV},3} = 0$ independent of the system states, the constraint will never become inactive. As x_3 is monotonically increasing, the lower constraint c_{lo} will never become active. The overall control u^* for this system is therefore given by

$$
u^*(t) = \begin{cases} 0 & , c_{\text{up}} > 0 \\ u_{\text{path}} & , c_{\text{up}} = 0 \end{cases} \tag{A4}
$$

B Steady-state analysis of the PV erythropoiesis model

Calculation of system's steady-state

Here, we present the calculation of the steady state for the extended erythropoiesis model (16) with regard to PV. Let B_{PV} denote the steady state of x_3. Then, $\bar{x} = (\bar{x}_1, \bar{x}_2, B_{\text{PV}})$ must be found, such that $\dot{x} = 0$. Using the structure

$$
\bar{x} := (\bar{x}_1, \bar{x}_2, \bar{x}_3) = \left(\frac{\alpha}{k_1}, \frac{\alpha}{k_2}, 1\right) \cdot B_{\text{PV}}, \tag{A5}
$$

the conditions $\dot{x}_2 = 0$ and $\dot{x}_3 = 0$ are already fulfilled. We use the right-hand side of x_1 with $X_0 = \alpha \cdot B$ to find

$$
\begin{aligned}
0 &\overset{!}{=} \beta \cdot (X_0 - k_1 \cdot \bar{x}_1) + (1 - \lambda_{\text{PV}}) \cdot Fb(B_{\text{PV}}) \cdot \bar{x}_1 + \lambda_{\text{PV}} \cdot \gamma^* \cdot \bar{x}_1 \\
&= \beta \cdot (\alpha \cdot B - \alpha \cdot B_{\text{PV}}) + (1 - \lambda_{\text{PV}}) \cdot \gamma \cdot \left(1 - \frac{B_{\text{PV}}}{B}\right) \cdot \frac{\alpha}{k_1} \cdot B_{\text{PV}} + \lambda_{\text{PV}} \cdot \gamma^* \cdot \frac{\alpha}{k_1} \cdot B_{\text{PV}} \\
&= (B_{\text{PV}})^2 \cdot \left[-\frac{\alpha}{k_1} \cdot \frac{(1 - \lambda_{\text{PV}}) \cdot \gamma}{B}\right] + B_{\text{PV}} \cdot \left[\frac{\alpha}{k_1} \cdot \left(-\beta \cdot k_1 + (1 - \lambda_{\text{PV}}) \cdot \gamma + \lambda_{\text{PV}} \cdot \gamma^*\right)\right] + \alpha \cdot \beta \cdot B
\end{aligned}
$$

For $\lambda_{\text{PV}} \neq 1$, this is equivalent to

$$
0 = (B_{\text{PV}})^2 + Y \cdot B_{\text{PV}} + Z \tag{A6}
$$

where

$$Y = B \cdot \frac{\beta \cdot k_1 - (1-\lambda_{\text{PV}}) \cdot \gamma - \lambda_{\text{PV}} \cdot \gamma^*}{(1-\lambda_{\text{PV}}) \cdot \gamma}$$
$$Z = -B^2 \cdot \frac{\beta \cdot k_1}{(1-\lambda_{\text{PV}}) \cdot \gamma}$$

which results in

$$B^{PV} = -\frac{Y}{2} \pm \sqrt{\frac{Y^2}{4} - Z} \tag{A7}$$

As $Z < 0$, the square-root expression always exists, and the positive solution is the desired one formulated in Equation (17). For $\lambda_{\text{PV}} = 1$ the squared expression vanishes and it follows the second expression in Equation (1) as

$$B_{\text{PV}} = B \cdot \frac{\beta \cdot k_1}{\beta \cdot k_1 - \gamma^*} \tag{A8}$$

Continuity of system's steady state

We now show that when using $\gamma^* = \frac{\beta}{10}$, this function is also continuous in $\lambda_{\text{PV}} = 1$. To prove this, we first observe that, with $k_1 = \frac{1}{8}$, it holds that

$$B_{\text{PV}}(\lambda_{\text{PV}} = 1) = B \cdot \frac{\frac{\beta}{8}}{\frac{\beta}{8} - \frac{\beta}{10}} = 5 \cdot B \tag{A9}$$

Therefore, we must prove that

$$\lim_{\lambda_{\text{PV}} \nearrow 1} \frac{B_{\text{PV}}(\lambda_{\text{PV}})}{B} = \frac{B_{\text{PV}}(1)}{B} = 5 \tag{A10}$$

We also note that the expression for Y simplifies to

$$Y = B \cdot \frac{\frac{\beta}{8}\left(1 - \frac{4}{5} \cdot \lambda_{\text{PV}}\right) - (1-\lambda_{\text{PV}}) \cdot \gamma}{(1-\lambda_{\text{PV}}) \cdot \gamma} \tag{A11}$$

Using this in (A7) for $\lambda_{\text{PV}} < 1$ results in

$$
\begin{aligned}
\frac{B_{\text{PV}}(\lambda_{\text{PV}})}{B} &= -\frac{\frac{\beta}{8}\left(1-\frac{4}{5}\cdot\lambda_{\text{PV}}\right)-(1-\lambda_{\text{PV}})\cdot\gamma}{2\cdot(1-\lambda_{\text{PV}})\cdot\gamma} \\
&\quad+\sqrt{\left(\frac{\frac{\beta}{8}\left(1-\frac{4}{5}\cdot\lambda_{\text{PV}}\right)-(1-\lambda_{\text{PV}})\cdot\gamma}{2\cdot(1-\lambda_{\text{PV}})\cdot\gamma}\right)^2+\frac{\frac{\beta}{8}}{(1-\lambda_{\text{PV}})\cdot\gamma}} \\
&= -\frac{\frac{\beta}{8}\left(1-\frac{4}{5}\cdot\lambda_{\text{PV}}\right)-(1-\lambda_{\text{PV}})\cdot\gamma}{2\cdot(1-\lambda_{\text{PV}})\cdot\gamma} \\
&\quad+\sqrt{\frac{\frac{\beta^2}{64}\left(1-\frac{4}{5}\cdot\lambda_{\text{PV}}\right)^2+\frac{\beta}{4}\cdot\left(1+\frac{4}{5}\cdot\lambda_{\text{PV}}\right)\cdot(1-\lambda_{\text{PV}})\cdot\gamma+(1-\lambda_{\text{PV}})^2\cdot\gamma^2}{4\cdot(1-\lambda_{\text{PV}})^2\cdot\gamma^2}} \\
&= \frac{1}{2\cdot(1-\lambda_{\text{PV}})\cdot\gamma}\cdot\left[-\frac{\beta}{8}\cdot\left(1-\frac{4}{5}\cdot\lambda_{\text{PV}}\right)+(1-\lambda_{\text{PV}})\cdot\gamma\right. \\
&\quad\left.+\sqrt{\frac{\beta^2}{64}\left(1-\frac{4}{5}\cdot\lambda_{\text{PV}}\right)^2+\frac{\beta}{4}\cdot\left(1+\frac{4}{5}\cdot\lambda_{\text{PV}}\right)\cdot(1-\lambda_{\text{PV}})\cdot\gamma+(1-\lambda_{\text{PV}})^2\cdot\gamma^2}\right]
\end{aligned}
$$

60 Both the numerator and the denominator are zero when $\lambda_{\text{PV}} = 1$. Therefore, L'Hospital's rule can be
61 employed to find the desired limit:

$$
\begin{aligned}
\lim_{\lambda_{\text{PV}}\nearrow 1}\frac{B_{\text{PV}}(\lambda_{\text{PV}})}{B} &= \lim_{\lambda_{\text{PV}}\nearrow 1}\frac{1}{\frac{d}{d\lambda_{\text{PV}}}\left(2\cdot(1-\lambda_{\text{PV}})\cdot\gamma\right)}\cdot\frac{d}{d\lambda_{\text{PV}}}\left[-\frac{\beta}{8}\cdot\left(1-\frac{4}{5}\cdot\lambda_{\text{PV}}\right)+(1-\lambda_{\text{PV}})\cdot\gamma\right. \\
&\quad\left.+\sqrt{\frac{\beta^2}{64}\left(1-\frac{4}{5}\cdot\lambda_{\text{PV}}\right)^2+\frac{\beta}{4}\cdot\left(1+\frac{4}{5}\cdot\lambda_{\text{PV}}\right)\cdot(1-\lambda_{\text{PV}})\cdot\gamma+(1-\lambda_{\text{PV}})^2\cdot\gamma^2}\right] \\
&= \lim_{\lambda_{\text{PV}}\nearrow 1}-\frac{1}{2\cdot\gamma}\cdot\left[-\frac{\beta}{10}-\gamma\right. \\
&\quad\left.+\frac{\frac{\beta^2}{40}\left(1-\frac{4}{5}\cdot\lambda_{\text{PV}}\right)+\frac{\beta}{5}\cdot(1-\lambda_{\text{PV}})\cdot\gamma-\frac{\beta}{4}\cdot\left(1+\frac{4}{5}\cdot\lambda_{\text{PV}}\right)\cdot\gamma-2\cdot(1-\lambda_{\text{PV}})\cdot\gamma^2}{2\cdot\sqrt{\frac{\beta^2}{64}\left(1-\frac{4}{5}\cdot\lambda_{\text{PV}}\right)^2+\frac{\beta}{4}\cdot\left(1+\frac{4}{5}\cdot\lambda_{\text{PV}}\right)\cdot(1-\lambda_{\text{PV}})\cdot\gamma+(1-\lambda_{\text{PV}})^2\cdot\gamma^2}}\right] \\
&= -\frac{1}{2\cdot\gamma}\cdot\left[-\frac{\beta}{10}-\gamma+\frac{\frac{\beta^2}{40}\cdot\frac{1}{5}+0-\frac{\beta}{4}\cdot\frac{9}{5}\cdot\gamma-0}{2\cdot\sqrt{\frac{\beta^2}{64}\cdot\frac{1}{25}+0+0}}\right] \\
&= -\frac{1}{2\cdot\gamma}\cdot\left[-\frac{\beta}{10}-\gamma+\frac{\beta}{10}-9\cdot\gamma\right] \\
&= 5
\end{aligned}
$$

REFERENCES

62 Bryson A, Ho YC. Applied Optimal Control (New York: Wiley) (1975).
